# Non-Selective Calcium Channel Blocker Bepridil Decreases Secondary Pathology in Mice after Photothrombotic Cortical Lesion

**DOI:** 10.1371/journal.pone.0060235

**Published:** 2013-03-26

**Authors:** Anu Lipsanen, Stefanie Flunkert, Kristina Kuptsova, Mikko Hiltunen, Manfred Windisch, Birgit Hutter-Paier, Jukka Jolkkonen

**Affiliations:** 1 Institute of Clinical Medicine-Neurology, University of Eastern Finland, Kuopio, Finland; 2 QPS Austria GmbH, Grambach, Austria; Oregon Health & Science University, United States of America

## Abstract

Experimental studies have identified a complex link between neurodegeneration, β-amyloid (Aβ) and calcium homeostasis. Here we asked whether early phase β-amyloid pathology in transgenic hAPP_SL_ mice exaggerates the ischemic lesion and remote secondary pathology in the thalamus, and whether a non-selective calcium channel blocker reduces these pathologies. Transgenic hAPP_SL_ (n = 33) and non-transgenic (n = 30) male mice (4–5 months) were subjected to unilateral cortical photothrombosis and treated with the non-selective calcium channel blocker bepridil (50 mg/kg, p.o., once a day) or vehicle for 28 days, starting administration 2 days after the operation. Animals were then perfused for histological analysis of infarct size, Aβ and calcium accumulation in the thalamus. Cortical photothrombosis resulted in a small infarct, which was associated with atypical Aβ and calcium accumulation in the ipsilateral thalamus. Transgenic mice had significantly smaller infarct volumes than non-transgenic littermates (*P*<0.05) and ischemia-induced rodent Aβ accumulation in the thalamus was lower in transgenic mice compared to non-transgenic mice (*P*<0.01). Bepridil decreased calcium load in the thalamus (*P*<0.01). The present data suggest less pronounced primary and secondary pathology in hAPP_SL_ transgenic mice after ischemic cortical injury. Bepridil particularly decreased calcium pathology in the thalamus following ischemia.

## Introduction

Alzheimer's disease (AD) and cerebrovascular disease are common pathologies in the elderly and are the most frequent contributors to neurodegeneration and cognitive impairment. It is now recognized that these pathologies often occur together and share common mechanisms [1]–[3].

Several studies have shown that AD and ischemic brain injury lead to altered amyloid precursor protein (APP) processing, β-amyloid (Aβ) accumulation [4]–[7], and increased neuroinflammation [8], [9] in cortical areas adjacent to the injury. More importantly, transgenic AD mice, such as APP_SWE_ and wtAPP751, show increased vulnerability to ischemic damage [10], [11]. In addition, exaggerated cognitive and motor deficits are observed in rats injected with Aβ_25_–_35_ before ischemia induced by endothelin-1 [12]–[14]. We have previously shown that APP and Aβ or their fragments accumulate in dense, plaque-like deposits in the thalamus of rats subjected to transient middle cerebral artery occlusion (MCAO) [15]. This coincides with delayed retrograde degeneration of thalamocortical connections and a robust inflammatory reaction [9]. Recently we also demonstrated that APP processing and the expression of Aβ-degrading enzymes are subsequently altered due to MCAO in the ipsilateral thalamus [16]. Interestingly, Aβ pathology shows an overlapping distribution pattern with aberrant calcium staining in the thalamus following MCAO [17]. Pharmacological evidence suggests a detrimental effect of thalamus pathology on behavioural functions [18].

Sarajärvi et al. [19] showed that chronic treatment of MCAO rats with the non-selective calcium channel blocker, bepridil, significantly decreases the levels of soluble Aβ_40_ and Aβ_42_, and calcium, in the ipsilateral thalamus as compared to vehicle treated rats, when treatment was started 2 days after operation. Since Aβ and calcium seem to be intimately linked in AD and cerebral ischemia [20]–[22], we subjected transgenic AD mice (hAPP_SL_) to a cortical photothrombotic lesion followed by daily treatment with a non-selective calcium channel blocker, bepridil (50 mg/kg). We hypothesized that the vulnerability of hAPP_SL_ transgenic mice to cortical infarction is markedly increased and that treatment with bepridil would reverse the secondary pathology in the thalamus. Contrary to our hypothesis, infarct volumes and secondary pathology were smaller in transgenic mice. In addition, bepridil treatment significantly decreased ischemia-induced calcium pathology in the ipsilateral thalamus.

## Materials and Methods

### Animals

**Figure 1:**
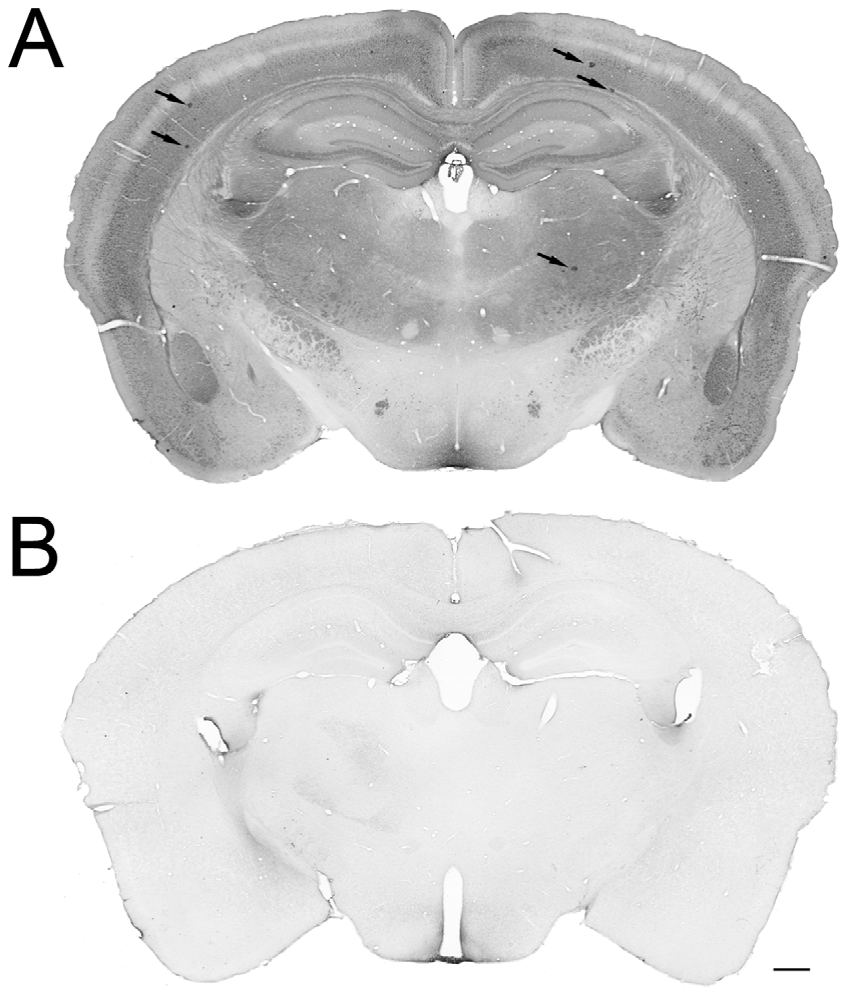
Confirmation of the transgenic Aβ phenotype. Human specific W0-2 staining against Aβ showed a relative high staining intensity in transgenic mice due to intracellular Aβ typical to young transgenic animals (A). A few plaques were observed (arrows). Only some non-specific staining was observed in non-transgenic mice due to degenerative processes (**B**). Scale bar: 400 µm.

A total of sixty-three male transgenic mice that overexpress hAPP_SL_ within a C57BL/6xDBA background, and non-transgenic littermates, were used in this study at an age of 4–5 months (QPS Austria GmbH, weight 25.9–46.7 g), when the first signs of plaques are usually observed [23], [24]. There was no human Aβ-positive staining in wildtype mice, thus confirming the phenotype (Figure 1B). The mice were housed in ventilated cages on standardized rodent bedding material (Rettenmaier®). Each cage contained a maximum of five mice. The mice had free access to food (Altromin®) and water throughout the experiment and were housed on a 12-hour light cycle in a temperature-controlled environment (24°C, humidity 40–70%). Animal studies conformed to the Austrian guidelines for the care and use of laboratory animals and were approved by the Styrian Government, Austria (FA10A-78 Jo 86 2011) and conducted in accordance with the guidelines set by the European Community Council Directives 86/609/EEC. All efforts were made to minimize the number of animals used and to ensure their welfare.

### Photothrombotic Cortical Lesion

**Figure 2:**
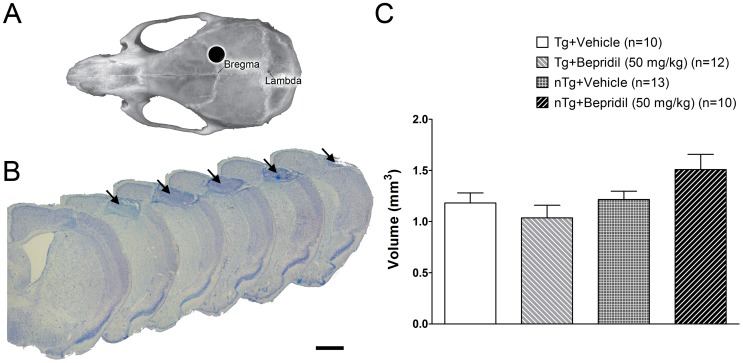
Rose Bengal induced cortical photothrombosis. (**A**) The cold light was positioned to illuminate the skull 2.4 mm right from Bregma. (**B**) Nissl-stained sections show a typical photothrombotic lesion in the right sensorimotor cortex (arrows). (**C**) The genotype had a significant effect on lesion size (two-way ANOVA, *P*<0.05) with smaller lesions in transgenic mice. Values are presented as mean±s.e.m. Scale bar: 1 mm (**B**).

Animals were anesthetized with a mixture (10 ml/kg body weight) containing 0.5 ml Ketasol (100 mg/ml), 0.1 ml Xylasol (4 mg/ml) and 4.4 ml 0.9% NaCl. Anesthetized animals were mounted to a stereotactic frame (Stoelting) and the skull was exposed with an approximate 1 cm incision along the midline. Then the cold light source was placed on the skull over the right sensorimotor cortex (2.4 mm from Bregma; Figure 2A). Before illumination, Rose Bengal (50 mg/kg) in saline was given via the tail vein as a bolus. Then the cold light (diameter 1 mm) was switched on for 10 minutes. After operation, the skin was glued with VetGlue and the animals were treated for postoperative pain with 10 ml/kg body weight of Rimadyl (0.05 mg/ml) and the local painkiller Xylocain gel (2%, AstraZeneca).

### Drug Treatment and Study Design

Two days after operation the animals were divided randomly into treatment groups: transgenic hAPP_SL_ mice treated with vehicle (n = 14); transgenic hAPP_SL_ mice treated with bepridil (50 mg/kg) (n = 13); non-transgenic mice treated with vehicle (n = 15) and non-transgenic animals treated with bepridil (50 mg/kg) (n = 15). Bepridil (Sigma-Aldrich) was first dissolved in ethanol (final volume 5%) and then added to a mixture of polyethylene glycol (Macrogol 400, Fargon, 50%) and 0.9% NaCl (45%). Animals were treated for 28 days with either vehicle or bepridil once a day with treatment starting on postoperative day 2 to avoid interference with acute ischemic processes. Animals received 50 mg/kg body weight of bepridil or vehicle orally via gavage (3.33 ml/kg). The selected dose had already been shown to be effective in reducing thalamic Aβ/calcium pathology in MCAO rats [19].

### General Health

General health was tested by a modified Irwin test [25]. The measured physical characteristics were body weight, body temperature, existence of whiskers, constitution of the fur and the eyes, bites or other lesions, and conspicuities, sensorimotor reflexes and motor abilities.

### Histology

All animals were perfused on postoperative day 30, one day after the last drug administration. Deeply anesthetized animals were perfused with 0.9% NaCl and 4% paraformaldehyde in phosphate buffered saline (PBS). The brains were removed from the skull and postfixed for four hours in 4% paraformaldehyde in PBS. After postfixation, brain samples were cryoprotected in 30% sucrose, frozen in isopentane and stored at −80°C.

Frozen sections (35 µm) were cut with a sliding microtome and stored in a cryoprotectant tissue collection solution at −20°C. The accumulation of Aβ in the thalamus was examined using a rodent-specific primary antibody (rabbit anti-rodent Aβ3-16, #9151; Covance, USA) and human-specific primary antibody (mouse monoclonal anti-amyloid β, clone W0-2, Millipore, USA). Adjacent sections were pretreated for 30 minutes with hot (85°C) citrate buffer. After citrate buffer incubation, the sections were rinsed three times in Tris buffered saline with 0.5% Triton X-100 (TBS-T) (rodent Aβ staining TBS-T pH 7.6 and W0-2 pH 8.6). Sections were transferred to a solution containing the primary antibody (rodent specific Aβ at 1∶5,000, human specific at 1∶20,000) in TBS-T for overnight incubation on a shaker at room temperature (20°C) in the dark. Then the sections were rinsed three times in TBS-T and transferred to a solution containing the secondary antibody (goat anti-rabbit IgG*biotin 1∶500; Millipore, USA, and goat anti-mouse*biotin 1∶500; Vector, USA). After 2 h, the sections were rinsed three times with TBS-T and transferred to a solution containing mouse ExtrAvidin (Sigma-Aldrich, USA), and finally incubated for 3 minutes with diaminobenzidine (DAB) [15]. Sections from aged APdE9 mice were used as a positive control.

For double immunofluorescence staining, sections were pretreated as described above and then incubated overnight with a mixture of primary antibodies; rabbit anti-rodent Aβ3-16 (1∶2,000, Covance, USA) and anti-mouse NeuN (1∶2,000, Millipore, Temecula, CA, USA) or anti-mouse anti-GFAP (1∶1,000, Sigma, St. Louis, USA). After rinsing in TBS-T, sections were incubated with secondary antibodies Alexa 488-conjugated goat anti-rabbit IgG (1∶250, Invitrogen, Carlsbad, CA, USA) and Alexa 594-conjugated goat anti-mouse IgG (1∶250, Invitrogen, Carlsbad, CA, USA). Finally, sections were mounted in mounting medium containing DAPI (Vectashield, Vector, USA). Images were captured using a Zeiss Axio Imager M2 microscope fitted with a camera, AxioCam MRm.

Calcium was stained with the Alizarin red method [17]. Sections were mounted on gelatinized glass and immersed in 2% (w/v, distilled water, pH 4.1 to 4.3) Alizarin Red (Merck, Germany) for 30 seconds followed by a rinse in distilled water. Sections were dehydrated with acetone and xylene and coverslipped with Depex.

### Quantification of Aβ and Calcium Accumulation in the Thalamus After Cortical Lesion

Images from stained sections were taken with an Olympus BX40 microscope and an Olympus digital camera DP50-CU (Japan). From each image, the thalamus was manually outlined (Photoshop CS3) and the areas of interest were automatically analyzed by using the ‘magic wand’ tool to outline the specific colour of staining from the background. Measurements were performed from two replicates and the mean values were used for statistical analysis.

### Assessment of Infarct Volumes

Infarct volumes were measured using ImageJ from Nissl-stained sections collected at 0.175–0.245 mm intervals. The mean area of tissue damage between two sequential sections was multiplied by the distance between the two sections, and these values (mm^2^) were summed up to receive total infarct volume (mm^3^). The researcher was blinded to the study design for all analyses.

### Statistical Analysis

SPSS Statistics software (Version 19) was used for statistical analyses. Body weight was analyzed using repeated measures analysis of variance (ANOVA). The effects of genotype and treatment on infarct volumes, Aβ and calcium staining were analyzed using two-way ANOVA followed by Student's t-tests if needed.

## Results

### Mortality and Exclusion of Mice

From 63 operated mice, 18 died or were excluded (71.4%). From these 18 animals, three animals were sacrificed right after the operation, three were euthanized after the operation due to unsuccessful intravenous injection, six animals were found dead during the follow-up and six animals were excluded from this study because they had no lesion in the cortex.

During the course of the study animals were observed daily to detect any physical signs as a result of the lesion or the treatment, and body weights were measured daily. The surgery did not cause weight loss in the animals. Statistical analysis by repeated measures ANOVA revealed that the genotype had a significant effect on body weight (*P*<0.001). The weight of non-transgenic mice was higher compared to transgenic mice before operation and then throughout the follow-up (Student's t-test, *P*<0.001). There was also a significant time and treatment interaction (*P*<0.05) indicating that bepridil-treated mice lost weight during the follow-up.

General health and motor function tests after the operation as measured by the Irwin test did not reveal differences between the groups.

### Lesion Volumes

Lesion size in the cortex was measured from Nissl-stained sections after completion of the treatment (Figure 2C). The lesion was highly limited to the sensorimotor cortex with no evidence of damage to the striatum (Figure 2B). Genotype had a significant effect on lesion size (two-way ANOVA, *P*<0.05). Infarct volumes were smaller in transgenic mice compared to non-transgenic mice (Student's t-test, *P*<0.05).

### Rodent *A*β Staining in the Thalamus Following Cortical Lesion

**Figure 3:**
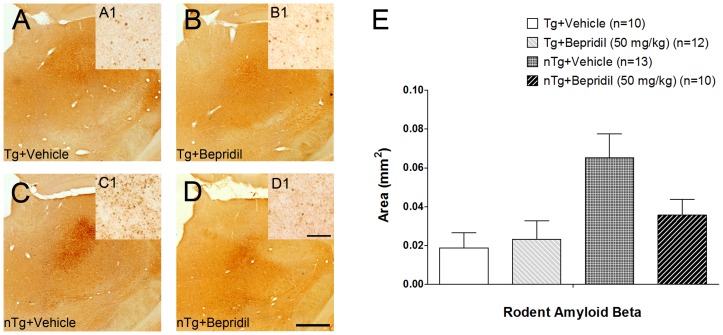
Rodent β-amyloid (Aβ) staining following cortical photothrombosis. Digital photomicrographs showed atypical, Aβ deposits in the thalamus ipsilateral to the cortical lesion (**A–D**). The inserts **A1–D1** at higher magnification are taken from the same brain sections. The genotype had a significant effect on rodent Aβ load (two-way ANOVA, *P*<0.05) being more pronounced in non-transgenic mice (**E**). Values are presented as mean±s.e.m. Scale bar: 500 µm (**A–D**), 20 µm (**A1–D1**).

**Figure 4:**
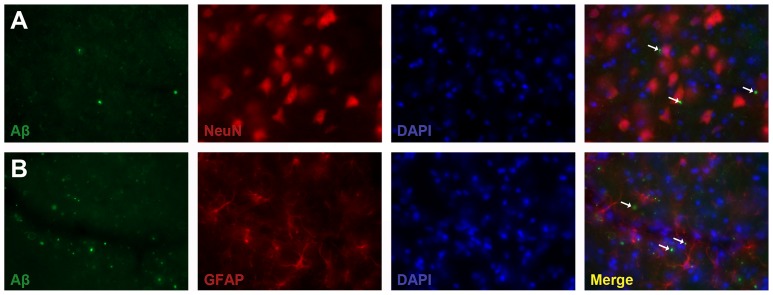
Cellular localization of rodent Aβ deposits in the thalamus following cortical photothrombosis. Double immunofluorescence staining for rodent Aβ and NeuN **(A)** or rodent Aβ and GFAP **(B)** did not show co-localization in AD transgenic mice (arrows).

A rodent specific Aβ antibody was used to reveal the endogenous amyloid deposits in the thalamus after cortical photothrombosis. Rodent Aβ deposits were observed only in the ipsilateral posterior nucleus in lesioned mice (Figure 3A–D), which is connected to the primary lesion in the cortex [26]. Aβ staining varied from diffuse staining to small granules (Figure 3A1–D1), but no plaque-like deposits were observed. The genotype had a significant effect on Aβ load in the thalamus (two-way ANOVA, *P*<0.01). Aβ accumulation in the thalamus after cortical lesion was more pronounced in non-transgenic mice (Student's t-test, *P*<0.01) (Figure 3). Double immunofluorescence staining for rodent Aβ and NeuN (neurons) or GFAP (astrocytes) in the thalamus after cortical photothrombosis did not show co-labeling (Figure 4).

### Human *A*β Staining in the Thalamus Following Cortical Lesion

W0-2 staining was used to stain human Aβ. Intracellular Aβ was observed in transgenic mice, which was most likely due to the young age of the animals (Figure 1A). Another human Aβ antibody 6E10 gave exactly the same staining pattern (data not shown). Lesion-induced human Aβ deposits were found in the thalamus, but only in a low quantity that was not reliably sufficient to measure from intracellular staining. There were only a few plaques in the cortex (1–5 plaques, 0.002 mm^2^) in transgenic mice. The area of plaques in the hemisphere ipsilateral to the lesion was not increased by cortical damage.

### Calcium Accumulation in the Thalamus Following Cortical Lesion

**Figure 5:**
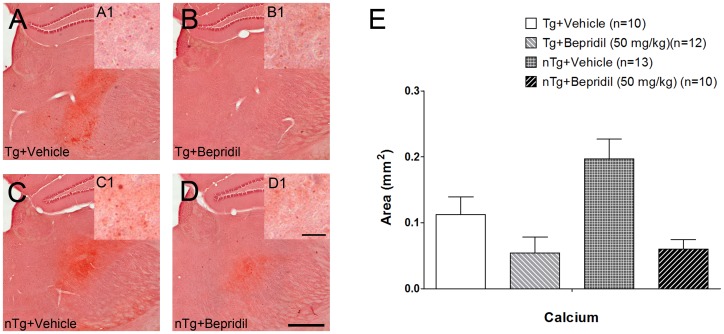
Calcium staining (Alizarin Red) following cortical photothrombosis. Digital photomicrographs showed atypical, calcium deposits in the thalamus ipsilateral to the cortical lesion (**A–D**). The inserts **A1–D1** at higher magnification are taken from the same brain sections. Treatment had a significant effect on calcium accumulation (two-way ANOVA, *P*<0.001) (**E**). Values are presented as mean±s.e.m. Scale bar: 500 µm (**A–D**), 20 µm (**A1–D1**).

Consistent with our previous studies [16], [17], Alizarin Red staining showed increased calcium accumulation from variable sizes in the ipsilateral thalamus after cortical lesion (Figure 5A–D). The size and location of the calcium deposits was similar to the Aβ staining from the adjacent section. There was a significant treatment effect in calcium accumulation (two-way ANOVA, *P*<0.01). However, there was no treatment and genotype interaction, indicating that transgenic and non-transgenic mice were similarly affected by bepridil treatment (Figure 5E).

## Discussion

Experimental studies have identified a complex link between neurodegeneration, β-amyloid (Aβ) and calcium homeostasis [20]–[22]. Therefore, the aim of the present study was to further examine this interaction in an AD mouse model.

Aβ, particularly when aggregated in oligomers, is neurotoxic [27] and when present it can exaggerate primary ischemic lesions [11]. Given this, we were surprised to see that the lesion size was even smaller in hAPP_SL_ transgenic mice compared to non-transgenic littermates. However, this is in line with our previous data following transient MCAO that shows significantly smaller infarct volumes in *hAPP695* rats compared to their littermates [28]. There is increasing evidence that APP or APP-derived peptides may have neurotrophic and neuroprotective functions *in vitro*, at least at certain concentrations [29], [30], [31]. Although *in vivo* evidence for a protective role of APP is sparse**,** the secreted N-terminal APP fragment, sAPPα, has been implicated in neuroprotection. Intracerebroventricular (i.c.v.) injection of sAPP has been shown to protect against ischemic brain injury in rats [32]. Similarly, i.c.v. injection of sAPPα or sAPPα domains improves motor and cognitive recovery following traumatic brain injury in rats [33], [34]. Thus, it is plausable that cortical lesion or trauma results in an immediate increase in processing and maturation of APP in the cortical neurons [19], which leads to generation of neuroprotective sAPPα through non-amyloid processing of APP to prevent neuronal damage. The exact neuroprotective mechanism is not known, but sAPPα may be able to buffer released Ca^2+^ [35] and trigger several neuroprotective signaling pathways, such as NF-κB, enhance the expression of downstream target genes such as MnSOD, and antagonize stress-induced pro-death pathways such as JNK/c-Jun (see [36].)

Remote areas such as the thalamus that are connected to the cortical lesion are affected due to delayed retrograde degeneration, which is followed by complex pathology [18], [37]. Previously we have shown that initial diffuse APP and Aβ staining transform into dense, plaque-like deposits in the ventroposterior lateral and ventroposterior medial nuclei (VPL/VPM) in MCAO rats [15]. Here we showed that a small lesion in the motor cortex produced rodent Aβ accumulation in the posterior nucleus that is connected to the primary lesion [26]. Accumulation was more pronounced in non-transgenic mice, again indicating that APP-derived peptides may mitigate secondary pathology in transgenic mice by reducing excitotoxicity (e.g., free radical production, buffering calcium overload) [35]. In addition, because of the small lesion without subcortical involvement, the Aβ pathology was less severe and varied from diffuse staining to small granules. No dense, plaque-like deposits were observed as seen previously in MCAO rats [15]. This allowed us to study the possible cellular localization of rodent Aβ deposits in the thalamus. Based on double immunohistochemistry it was clear that Aβ deposits were not localized in neurons (NeuN-positive) or astrocytes (GFAP-positive), which strongly suggests that the deposits are extracellular at least 30 days after cortical photothrombosis. Recently, autophagosomes were shown to accumulate within thalamic neurons after cerebral ischemia, possibly contributing to neuronal death [38]. Since autophagosomes contain APP, Aβ and β-secretase (BACE) [39], it would be tempting to speculate that these vacuoles are released to the extracellular space after cell lysis, explaining the presence of granular Aβ deposits in the affected thalamic nuclei. However, exosomes may as well be involved in recycling APP or Aβ and cellular constituents by releasing them from the cell [40].

There was also a sign of human Aβ positive deposits in the thalamus after ischemia, but at a level that was not technically possible to measure from intracellular staining. Six-month-old hAPP_SL_ transgenic mice express the transgene in relative high level (Figure 1A) [23], [24] not explaining low accumulation of human Aβ in the thalamus. Rather this could indicate that human APP and endogenous APP are processed and/or cleared in a different manner in the thalamus, a notion consistent with our human *post mortem* study [41] and data from non-human primates subjected to transient MCAO (Lipsanen et al., unpublished data), which did not show human Aβ aggregations in the thalamus after cerebrovascular lesions. The area of Aβ plaques in the cortex was low in transgenic mice but there was no evidence for increase in Aβ plaques in the hemisphere ipsilateral to the lesion.

Thalamic pathology after cerebral ischemia includes impaired calcium homeostasis [42]. Accumulation of calcium granules of variable size was observed also in mice subjected to photothrombosis. Interestingly, calcium is known to aggregate in the thalamus with an overlapping distribution pattern with Aβ [17] and calcium phosphate precipitates can potentially activate autophagy [43]. Thus, calcium phosphate might be the trigger leading to delayed neuronal death through autophagosome accumulation in the thalamus after ischemia [38]. Electron microscopy at early time points after cerebral ischemia is needed to elucidate this process in detail.

Previously we have shown that bepridil treatment decreases soluble Aβ_40_ and Aβ_42_, and calcium concentrations, in the thalamus in MCAO rats [19]. To test whether bepridil is effective in another ischemia model and species, half of the mice were treated with the non-selective calcium channel blocker bepridil. Consistent with the rat data [19], bepridil decreased ischemia-induced calcium load in the thalamus. Although bepridil may exert its effects on intracellular calcium through multiple mechanisms, the data from anoxic rat optic nerve studies suggest that bepridil also prevents calcium entry through the reverse operation of the sodium-calcium exchanger in addition to the inhibition of voltage-gated calcium channels [44]. There was only a non-significant decrease in the Aβ load in the thalamus and in the number of cortical plaques due to bepridil treatment.

In conclusion, the present data show less pronounced primary and secondary pathology in non-symptomatic transgenic hAPP_SL_ mice after cortical injury, suggesting an initial neuroprotective role for APP. The remote calcium pathology in the thalamus was effectively decreased by treatment with bepridil. Thus, bepridil is an attractive drug candidate for the treatment of neurodegenerative diseases involving calcium dysregulation.
